# Polymorphisms of Chicken TLR3 and 7 in Different Breeds

**DOI:** 10.1371/journal.pone.0119967

**Published:** 2015-03-17

**Authors:** Wenke Ruan, Jian An, Yanhua Wu

**Affiliations:** 1 College of Animal Science and Technology, Beijing University of Agriculture, Beijing 102206, China; 2 Department of Microbiology, School of Basic Medical Sciences, Capital Medical University, Beijing 100069, China; CSIRO, AUSTRALIA

## Abstract

Toll-like receptors (TLRs) mediate immune responses via the recognition of pathogen-associated molecular patterns (PAMPs), thus playing important roles in host defense. Among the chicken (Ch) TLR family, ChTLR3 and 7 have been shown to recognize viral RNA. In our earlier studies, we have reported polymorphisms of TLR1, 2, 4, 5, 15 and 21. In the present study, we amplified TLR3 and 7 genes from different chicken breeds and analyzed their sequences. We identified 7 amino acid polymorphism sites in ChTLR3 with 6 outer part sites and 1 inner part site, and 4 amino acid polymorphism sites in ChTLR7 with 3 outer part sites and 1 inner part site. These results demonstrate that ChTLR genes are polymorphic among different chicken breeds, suggesting a varied resistance across numerous chicken breeds. This information might help improve chicken health by breeding and vaccination.

## Introduction

Viral diseases pose serious threats to the poultry industry. Frequent variation in virus is the major cause of immunity failure. This causes outbreaks of viral diseases in poultry. Therefore, improving genetic resistance to viruses may provide an alternative control measure against viral infections.

Toll-like receptors (TLRs) play a crucial role in the host innate immune response via the recognition of pathogen-associated molecular patterns (PAMPs) [1]. Ten members of the chicken (Ch) TLR family have been identified, including TLR1 (types 1 and 2), TLR2 (types 1 and 2), TLR3, 4, 5, 7, 15, and 21 [2]. ChTLR1 type 1 and type 2, as well as ChTLR2 type 1 and type 2, cooperatively recognize bacterial peptidoglycans (PGN), lipopolysaccharides (LPS) and lipid, which constitute the bacterial cell wall components. ChTLR4 recognizes bacterial LPS [3], while ChTLR5 recognizes the protein components of bacterial flagellum [4]. ChTLR15 is unique to avian and recognizes a yeast-derived components [5]. ChTLR21 recognizes microbial DNA and synthetic oligodeoxynucleotides (ODN) containing CpG dinucleotides [6]. Additionally, ChTLR3 and ChTLR7 recognize viral double-stranded RNA, single-stranded RNA and the artificial agonist poly(I:C) [2,7]. ChTLR1, 2, 4, 5, and 15 are located on the cell surface, while ChTLR3, 7, and 21 are located in the cytoplasm [2].

Our previous studies have demonstrated polymorphisms in ChTLR1, 2, 4, 5, 15, and 21 [8,9,10,11]. In the present study, we reported polymorphisms in ChTLR3 and 7 in a panel of chicken breeds and focused on ChTLR polymorphisms involved in viral recognition.

## Materials and Methods

We chose nine different chicken breeds, including the White Leghorn (WL) layer chicken (white shell layer), the Hy-Line variety (HL) Brown layer chicken (brown shell layer), the China Beijing White 939 (BW) layer chicken (pink shell layer), the China Nongda No. 3 (NN3) layer chicken (pink shell layer), the Luhua (LH) meat chicken (which originated from the American Plymouth Rock variety), the Royal (RY) meat chicken (originated from the United Kingdom), the White-Feather Silky (WS) dual chicken (which is characterized by white feathers and black bones), the China Beijing Fatty (BF) meat chicken and the Laiwu Black (LB) meat chicken (which is characterized by its shape). The chicken breeds used in this study were obtained from the Beijing Poultry Breeding Center (Beijing, China). In the present study, four individuals were sacrificed for the experiment from each breed. All procedures were approved by the Animal Care and Use Committee of Beijing University of Agriculture (Beijing, China).

Total RNA was obtained from spleens using TRIzol (Invitrogen, Carlsbad, CA), following the manufacturer’s instructions, and used to synthesize total cDNA. Briefly, total 20 μl of reaction mixture containing 5 μg of total RNA, 0.5 μg of oligo dT primer (16–18 mer), 40 U of RNasin, 1,000 μM of dNTP mix, 10 mM of DTT and 5 U of M-MLV reverse transcriptase (Promega, Madison, WI) in 1× reverse transcriptase buffer was incubated at 37°C for 1 h. The successful cDNA synthesis was confirmed by amplifying the β-actin amplicon by PCR. The two pairs of primers employed to amplify the full open reading frames (ORF) of TLR3 and TLR7 were designed according to the consensus sequences of TLR3 and 7 mRNA of *Gallus gallus*: 5’- ACACAGGATGTTTACATGCGATT-3’ (forward sequence) and 5’- TTCCTGCACCATGGATCCTGTAAGA-3’(reverse sequence) for ChTLR3 (NM 001011691), 5’- GTACATCATGCAAGGACGTCAAAT-3’ (forward sequence) and 5’- CAGTTTCCTGGAGAAGTTTGTTGTA-3’ (reverse sequence) for ChTLR7 (NM 001011688). The reaction conditions of the PCR were the same for both ChTLR3 and 7. Briefly, total 25 μl of PCR reaction mixture containing 50 pmol for both forward and reverse primers, 2 μl Template cDNA, 200 μM each of dNTP mix and 2.5 U *Pfu* DNA polymerase (Promega) in 1× *Pfu* DNA polymerase buffer was incubated under the following conditions: initial denaturation at 94°C for 3 min, 32 cycles at 94°C for 30 s, annealing at 56°C- 60°C for 30 s, and extension at 72°C for 5 min, followed by a final extension at 72°C for 7 min. PCR amplicons were verified by 1% agarose gel electrophoresis, then ligated into pEASY-Blunt simple cloning vector (TransGen, Beijing, China). Recombinant plasmids were characterized by PCR using gene specific and vector primer pairs. Recombinant plasmids with ChTLR3 and 7 were sequenced from both ends using an ABI 3730XL sequencer (Sinogenomax co. ltd, Beijing, China).

The coding sequences for ChTLR3 and 7 have been deposited in GenBank. The accession numbers are: ChTLR3: KM486590 (BW), KM486591 (WL), KM486592 (RY), KM486593 (LB), KM486594 (HL), KM486595 (LH), KM486596 (NN3), KM486597 (WS), and KM486598 (BF); ChTLR7: KM486599 (BW), KM486600 (WL), KM486601 (RY), KM486602 (LB), KM486603 (HL), KM486604 (LH), KM486605 (NN3), KM486606 (WS), and KM486607 (BF). The nucleotide and amino acid sequences of ChTLR3 or 7 were aligned with MegAlign analysis software (DNAstar Inc., Madison, USA) and compared to the reference sequences ChTLR3 and 7 (Genbank accession no. NM 001011691 and NM 001011688). The extracellular, transmembrane, and inner part of these protein sequences were predicted with the analysis tools provided on the web (http://smart.embl-heidelberg.de). The crystal structure of the ChTLR outer part was predicted by the CPHmodels 3.0 Server (www.cbs.dtu.dk). The amino acids corresponding to non-synonymous SNPs in the outer part were visualized by PyMOL v0.99.

## Results and Discussion

Different chicken breeds display varied sequence patterns in their TLRs. The amino acid sequence analysis showed that there are 7 and 4 sites of varied amino acid sequences in ChTLR3 and 7, respectively. In ChTLR3, 6 polymorphic sites (D14V, R345S, G362E, R459K, A540V, and A649V) are located in the outer part, while 1 polymorphic site (T713S) is located in the inner part (Table 1). The TLR3 genes are associated with human susceptibility to viral infections. Additionally, it has been reported that a polymorphism in TLR3 gene was associated with susceptibility to hepatitis B virus infection, subacute sclerosing panencephalitis, chronic hepatitis C and idiopathic pulmonary fibrosis [12,13,14,15]. In ChTLR7, 3 polymorphic sites (V91L, S135T and P669S) are located in the outer part, while 1 polymorphic site (V876M) is located in the inner part (Table 2). In a previous study, it has been shown that polymorphisms in TLR7, which is similar to TLR3 in its ability to recognize viral RNA in humans, are associated with human susceptibility to viral disease, such as chronic hepatitis C [16]. TLR3 and 7 polymorphisms may have a profound influence on host responses to a wide range of viruses and therefore may be associated with viral disease resistance or susceptibility [17].

**Table 1:** Chicken TLR3 polymorphic sites in different breeds.

Position(AA)^1^	Structural character	Majority AA (codon)	Polymorphic AA (codon)	Breed^2^	Synonymous/nonsynonymous SNP^3^
14		D(GAT)	V(GTT)	HL, WS	No
165	Leucine-rich repeats	A(GCG)	A(GCA)	HL, LB, LH, NN3	Yes
190		E(GAG)	E(GAA)	BF, BW, HL, LB, WL,	Yes
334	Leucine-rich repeats	T(ACT)	T(ACC)	LH, NN3	Yes
345	Leucine-rich repeats	R(AGG)	S(AGC)	BW, HL, NN3, WL	No
362	Leucine-rich repeats	G(GGG)	E(GAG)	BF, BW, HL, LB, LH, NN3, RY, WL, WS	No
459	Leucine-rich repeats	R(AGG)	K(AAG)	BF, BW, HL, LH, NN3, RY, WL	No
540	Leucine-rich repeats	A(GCG)	V(GTG)	LB, WS	No
617	Leucine-rich repeats	P(CCG)	P(CCA)	HL, LB	Yes
649		A(GCT)	V(GTT)	BW, HL, LH, NN3, RY, WL, WS	No
694	TIR domain	D(GAC)	D(GAT)	HL, NN3	Yes
713	TIR domain	T(ACT)	S(TCT)	BW, HL, LH, NN3, RY, WL	No
819	TIR domain	V(GTC)	V(GTT)	BW, HL, WL	Yes

^1^Aa = Amino acid

^2^ BF = Beijing Fatty chicken, BW = Beijing White 939 chicken, HL = Hy-Linevarietybrown chicken, LB = Laiwu Black chicken, LH = Luhua chicken, NN3 = Nongda No.3 chicken, RY = Royal chicken, WL = White Leghorn chicken, WS = White-Feather Silky chicken, referred sequence = Gallus gallus TLR3 NM_001011691.

^3^ Synonymous (n = 6) and nonsynonymous (n = 7) substitution.

**Table 2:** Chicken TLR7 polymorphic sites in different breeds.

Position(AA)^1^	Structural character	Majority AA (codon)	Polymorphic AA (codon)	Breed^2^	Synonymous/nonsynonymous SNP ^3^
91		V(GTA)	L(GCA)	RY, WS	No
135	Leucine-rich repeats	S(TCA)	T(ACA)	WS	No
669	Leucine-rich repeats	P(CCT)	S(TCT)	HL, RY, WL, WS	No
876		V(GTG)	M(ATG)	BF, BW, HL, LB, LH, NN3, WL	No
924	TIR domain	E(GAA)	E(GAG)	BF, BW, HL, LB, LH, NN3, RY, WL	Yes

^1^Aa = Amino acid

^2^ BF = Beijing Fatty chicken, BW = Beijing White 939 chicken, HL = Hy-Linevarietybrown chicken, LB = Laiwu Black chicken, LH = Luhua chicken, NN3 = Nongda No.3 chicken, RY = Royal chicken, WL = White Leghorn chicken, WS = White-Feather Silky chicken, referred sequence = Gallus gallus TLR7 NM 001011688.

^3^ Synonymous (n = 1) and nonsynonymous (n = 4) substitution.

Because the TLR8 gene in chickens has been disrupted, therefore ChTLR 3 and 7 are involved in the recognition of viral RNA in chicken. For instance, several studies have shown that the expression of ChTLR 3 and 7 are associated with Marek's disease virus, Newcastle disease virus, infectious bursa disease virus, and avian influenza virus infection [18,19,20,21]. Thus, those polymorphic sites found in ChTLR3 and 7 may be involved in the resistance or susceptibility of chickens to above viral infection.

TLRs are type I trans-membrane proteins with a large outer part composed of leucine-rich repeats (LRRs), a trans-membrane domain and a cytoplasmic Toll/interleukin-1 receptor (TIR) domain. LRRs are involved in PAMPs recognition. Because LRRs and LRRCTs are ligand binding domains [1,22], the variation of amino acids in LRR domains in ChTLR3 (R345S, G362E, R459K, and A540V) and ChTLR7 (S135T and P669S) might affect PAMP recognition by ChTLRs. TIR domain shares similarity with the interleukin 1 (IL-1) receptor, which is highly conserved and interacts with adapter proteins such as myeloid differentiation factor (MyD88) [1]. However, we found mutations in amino acid sites of the TIR domain of ChTLR3 (T713S) in Beijing Fatty chickens, White-Feather Silky chickens, Laiwu Black chickens which are all Chinese native breeds, which might be regional differences in the evolution of animals [23].

Differences in susceptibility to avian influenza virus, avian leukosis virus, infectious bronchitis virus, laryngotracheitis virus, and infectious bursal virus among breeds of chickens have been described [24,25,26,27,28,29]. It has been shown that the White Leghorn chickens appear to be some resistance to H7N1 highly pathogenic avian influenza virus [28]. However, the cause for the resistance of the breed to this viral disease is still to be clarified. As innate immunity is the first line of host defense, it is possible that the allelic variation in ChTLR3 and 7 may be connected with host defense. In the present study, we found 3 polymorphic sites in ChTLR3 (R345S, G362E, and R459K) and 2 sites in ChTLR7 (P669S and V876M) in White Leghorn chickens. Among the chicken breeds used in the present experiments, White-Feather Silky chickens would be a good model to study the disease resistance in chicken [30]. This breed has almost all polymorphic sites (D14V, E362G, K459R, A540V, A649V, and T713S in ChTLR3, V91L, S135T, P669S, and V876M in ChTLR7). These findings may provide some clues towards the understanding of the resistance of different chicken breeds to viral diseases. In addition, the polymorphic site R362E in ChTLR3 exists in all chicken breeds determined in the present study, suggesting that the evolution processes appear to have occurred in these breeds. These data also implicate that the heterogeneity of chicken TLR proteins of various breeds is preserved in chicken breeds, which is possibly associated with breeding and selection. The further studies need to be done to verify the effects of these mutations.

**Fig 1:**
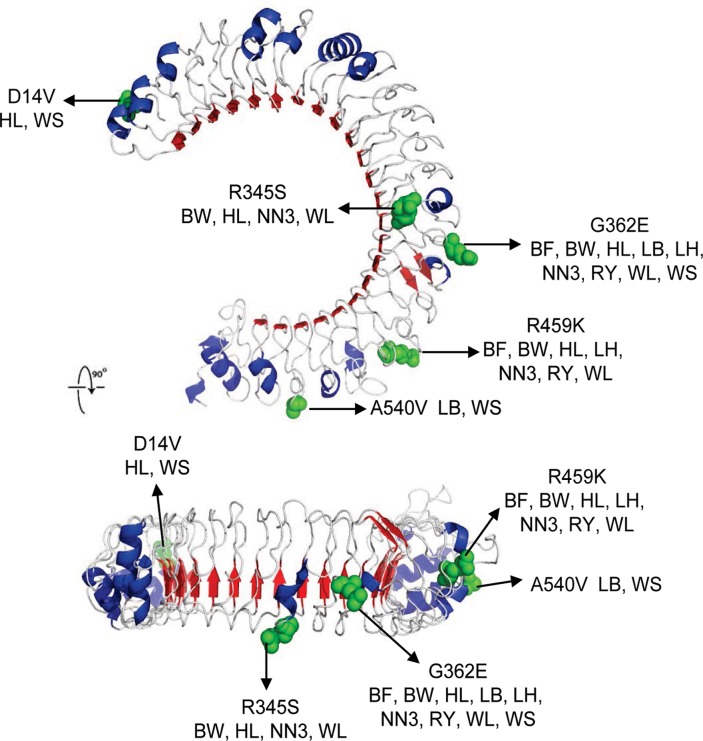
Visualization of amino acids corresponding to nonsynonymous single nucleotide polymorphism in extracellular domain of chicken TLR3 based on the protein structure predicted by CPHmodels 3.0 Server (www.cbs.dtu.dk). BF = Beijing Fatty chicken, BW = Beijing White 939 chicken, HL = Hy-Linevarietybrown chicken, LB = Laiwu Black chicken, LH = Luhua chicken, NN3 = Nongda No. 3 chicken, RY = Royal chicken, WL = White Leghorn chicken, WS = White-Feather Silky chicken.

**Fig 2:**
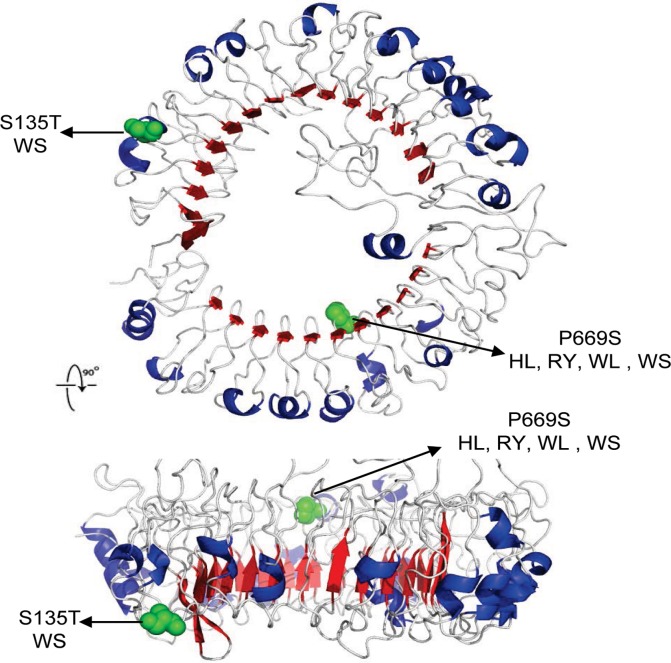
Visualization of amino acids corresponding to nonsynonymous single nucleotide polymorphism in extracellular domain of chicken TLR7 based on the protein structure predicted by CPHmodels 3.0 Server (www.cbs.dtu.dk). HL = Hy-Linevarietybrown chicken, RY = Royal chicken, WL = White Leghorn chicken, WS = White-Feather Silky chicken.

To put the observed polymorphic sites in a functional context, we visualized the variable amino acid positions in the three-dimensional structures of ChTLR3 and 7 outer part using the crystal structure predicted by the CPHmodels 3.0 Server (www.cbs.dtu.dk) (Figs. 1 and 2). It is likely that mutations of the external polymorphic sites may have a greater effect than that of the internal sites. Amino acid mutations D14V, R345S, G362E, R459K, and A540V are located in the external parts of ChTLR3 (Fig. 1), while amino acid mutation S135T is located in the external parts of ChTLR7 (Fig. 2). These polymorphic sites might influence PAMP recognition. It is possible that the differences in these amino acids cause subtle differences in the protein structure and thus alter the ligand-receptor binding.

In conclusion, we investigated polymorphic sites of ChTLR3 and 7 among various chicken breeds. Similar to our previous findings in ChTLR1, 2, 4, 5, 15, and 21, we found that there are 7 polymorphic sites in ChTLR3 and 4 polymorphic sites in ChTLR7, respectively. At the level of viral recognition, polymorphisms in ChTLR3 and 7 may be associated with resistance or susceptibility of chickens to viral infection. This basic information may improve the current understanding of variable resistance to viral diseases among different chicken breeds.
